# HURRICANE KATRINA’S LASTING IMPACT ON YOUNGER AND OLDER ADULTS: THE ROLE OF RELIGIOSITY AND POSTTRAUMATIC GROWTH

**DOI:** 10.1093/geroni/igad104.2667

**Published:** 2023-12-21

**Authors:** Katie Cherry, Marlene Friis, Piper Bordes, Kennedy Simon

**Affiliations:** Louisiana State University, Baton Rouge, Louisiana, United States; Tulane University, New Orleans, Louisiana, United States; Louisiana State University, Baton Rouge, Louisiana, United States; Louisiana State University, Baton Rouge, Louisiana, United States

## Abstract

In 2005, Hurricane Katrina devastated the U.S. Gulf Coast with catastrophic damage and substantial loss of life. In this study, we examined associations among psychosocial variables and mental health indicators in younger and older adults who lived in south Louisiana in August of 2005 when Katrina made landfall (M age = 51.7 years, SD = 13.03 years, age range: 32 to 78 years). All self-identified as African American / Black women. They were recruited through social media, flyers sent to churches and schools, and word of mouth referrals. On the first day of testing, participants completed sociodemographic, religiosity, and post-traumatic growth surveys and an adapted structured storm questionnaire (Cherry et al., 2011). On the second day of testing one week later, participants responded to open-ended questions on their Katrina experience, and they completed measures of post-traumatic stress disorder (PTSD) and depression. Correlation analyses revealed that age was inversely related to PTSD symptoms, which is compatible with an inoculation perspective on post-disaster psychological reactions. In addition, post-traumatic growth was positively correlated with religious beliefs and practices and religious coping, and negatively correlated with symptoms of PTSD and depression. Religious beliefs and practices were inversely correlated with PTSD symptoms and depression. Qualitative analyses of participants’ narrative responses indicated that some reflected on scripture and utilized prayer as a way to navigate their new lives in the years after Katrina. Implications of these data for understanding individual and community-level challenges in the years after exposure to devastating severe weather events are considered.

